# Properties of smooth pursuit and visual motion reaction time to second-order motion stimuli

**DOI:** 10.1371/journal.pone.0243430

**Published:** 2020-12-14

**Authors:** Takeshi Miyamoto, Kenichiro Miura, Tomohiro Kizuka, Seiji Ono

**Affiliations:** 1 Graduate School of Comprehensive Human Sciences, University of Tsukuba, Ibaraki, Japan; 2 Department of Pathology of Mental Diseases, National Institute of Mental Health, National Center of Neurology and Psychiatry, Tokyo, Japan; 3 Department of Integrative Brain Science, Graduate School of Medicine, Kyoto University, Kyoto, Japan; 4 Faculty of Health and Sport Sciences, University of Tsukuba, Tsukuba, Japan; University of Muenster, GERMANY

## Abstract

A large number of psychophysical and neurophysiological studies have demonstrated that smooth pursuit eye movements are tightly related to visual motion perception. This could be due to the fact that visual motion sensitive cortical areas such as meddle temporal (MT), medial superior temporal (MST) areas are involved in motion perception as well as pursuit initiation. Although the directional-discrimination and perceived target velocity tasks are used to evaluate visual motion perception, it is still uncertain whether the speed of visual motion perception, which is determined by visuomotor reaction time (RT) to a small target, is related to pursuit initiation. Therefore, we attempted to determine the relationship between pursuit latency/acceleration and the visual motion RT which was measured to the visual motion stimuli that moved leftward or rightward. The participants were instructed to fixate on a stationary target and press one of the buttons corresponding to the direction of target motion as soon as possible once the target starts to move. We applied five different visual motion stimuli including first- and second-order motion for smooth pursuit and visual motion RT tasks. It is well known that second-order motion induces lower retinal image motion, which elicits weaker responses in MT and MST compared to first-order motion stimuli. Our results showed that pursuit initiation including latency and initial eye acceleration were suppressed by second-order motion. In addition, second-order motion caused a delay in visual motion RT. The better performances in both pursuit initiation and visual motion RT were observed for first-order motion, whereas second-order (theta motion) induced remarkable deficits in both variables. Furthermore, significant Pearson’s correlation and within-subjects correlation coefficients were obtained between visual motion RT and pursuit latency/acceleration. Our findings support the suggestion that there is a common neuronal pathway involved in both pursuit initiation and the speed of visual motion perception.

## Introduction

When we look at a moving target, smooth pursuit eye movements ensure to hold the image of a moving target on or near the fovea in order to obtain clear vision. The control mechanism involved in smooth pursuit has been eagerly explored by the use of sub-human primates and single-unit recording. Smooth pursuit is driven by visual motion of the attended target on the retina and especially pursuit initiation is closely related to retinal image motion. Retinal image motion signals are transformed into pursuit commands and thereby smooth pursuit is initiated [1–3]. It is well established that the initial part of smooth pursuit, the open-loop period, reflects visual motion processing. Several studies have revealed that the initial part of smooth pursuit is driven by visual motion related signals from cortical areas including the middle temporal (MT) and medial superior temporal (MST) areas [4, 5]. In fact, Osborne and colleagues have demonstrated that the variability of pursuit initiation is mostly due to sensory errors in estimating target motion in monkeys [6, 7]. Besides, they have reported that almost all available directional information can be extracted from the first few spikes of the response of MT neuron, which is comparable with the pursuit initiation in terms of time scale [8].

A large number of psychophysical studies have shown that smooth pursuit is closely related to motion perception such as the direction-discrimination and perceived velocity of a moving target [9–12]. For instance, the steady-state smooth pursuit gain and the perceived target velocity are altered in association with the contrast of visual motion stimuli [13] and are in the same manner affected by type of random-dot kinematograms [14]. Moreover, a smooth pursuit adaptation paradigm leads to changes in not only smooth pursuit per se but also perceived target velocity [15]. Furthermore, during a isoluminant motion stimulus, both initial eye acceleration and perceived velocity are decreased compared to luminance-based stimuli which have comparable contrast [16]. The relevance between smooth pursuit and motion perception is plausible because the MT and MST play a pivotal role in motion perception as well as smooth pursuit [17, 18]. Furthermore, lesions of MT lead to selective deficit of smooth pursuit as well as motion perception in monkeys [19] and humans [20].

Although those studies have revealed that smooth pursuit is associated with motion perception by using the direction-discrimination task and a perceptual target velocity task, only a few studies have focused on the speed of visual motion perception [21]. Visual-motor reaction time (RT) is known as one of the common methods to evaluate speed of visuomotor processing which is the time lapse between the onset of the visual stimulus and the appearance of a motor response [22]. Previous studies have demonstrated that visual-motor RT is dependent on the speed of visual signal perception and sensory motor processing [23, 24]. Generally, an appearance of symbol or light as a visual stimulus is used in the visual-motor RT task [25, 26]. Ono and colleagues have applied the visual motion target to visual-motor RT task (visual motion RT) where a centered target moves toward right or left. The visual motion RT has shown that the horizontal asymmetry of initial eye acceleration is correlated with asymmetry of visual motion RT, suggesting the existence of a common neuronal pathway involved in both pursuit initiation and visual motion RT [21]. However, the relationship between altered pursuit initiation and visual motion RT remains largely unclear. Assuming that there is a common neuronal pathway, a factor that leads to reduction in pursuit initiation would induce delay of visual motion RT as well. Therefore, we attempted to apply different visual motion stimuli to visual motion RT tasks to clarify the agreement between pursuit initiation and visual motion RT.

Smooth pursuit eye movements are known to be induced by a visual stimulus which has not a primary luminance cue. The first-order motion is defined by the low level visual motion such as luminance, whereas the second-order motion is defined by the higher level visual motion such as contrast, flicker of motion [27–29]. A difference in visual motion stimuli affects the behavioral property of smooth pursuit eye movement, such as pursuit latency, initial eye acceleration and steady-state eye velocity [30–33]. Furthermore, neurons in area MT and MST that code for the direction of target motion show the weaker responses when the visual motion stimulus is formed by second-order motion compared to first-order motion [31, 33, 34]. Therefore, we hypothesized that the visual motion RT would be altered dependent on visual motion stimuli in association with pursuit initiation.

## Material and methods

To test our hypothesis, we set two separate experiments (smooth pursuit eye movement task and visual motion RT task) rather than a single experiment based on a methodological advantage. In the visual motion RT task, the participants were required to judge the visual motion direction after the target onset (see below for details). Such visual motion induces catch-up saccades after the onset of visual motion. Although the pre-saccadic pursuit initiation reflects the open-loop pursuit that is generated by retinal image motion [30], a large number of trials are needed to evaluate the pursuit initiation in the single visual motion RT task. Furthermore, a dual task of pursuit and pressing a button may inhibit pursuit initial responses compared to a single task of pursuit. Thus, we set another experiment focusing on pursuit initiation using a step-ramp paradigm [35]. In this paradigm, the target stepped in one direction and immediately moved at a constant speed in the other direction, which allows us to examine the initial pursuit response without occurrence of saccades and makes it possible to evaluate pursuit initiation by fewer trials compared to the single visual motion RT task.

### Participants

The participants were eight males and four females with a mean age of 23.3 years (range 22–25) and they reported having normal or corrected to normal vision and no known motor deficits. The participants were diagnosed neither as a stereoscopic problem nor strabismus. This study was conducted in accordance with the Declaration of Helsinki, and all protocols were approved by the Research Ethics Committee at the Faculty of Health and Sport Sciences, University of Tsukuba.

### Smooth pursuit eye movement task

**Fig 1:**
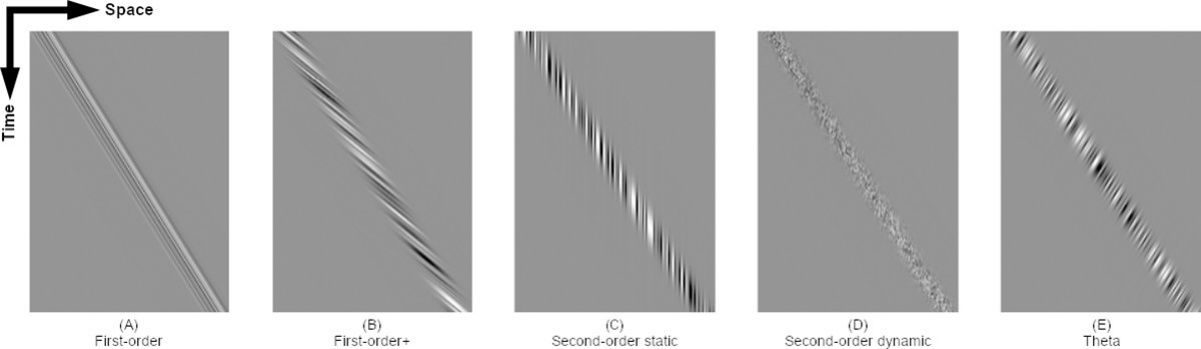
Space–time diagrams of five motion stimuli. (A) For the first-order motion target, both the Gaussian window and random dots texture moved in the same direction at the same speed. (B) For the first-order+ motion target, the Gaussian window and random dots texture moved in the same direction but random dots texture moved at twice the speed of the Gaussian window. (C) For the second-order static motion target, the Gaussian window moved over a static-background that consists of random dots texture. (D) For the second-order dynamic motion target, the Gaussian window moved over a dynamic-background where random dots texture was replaced on every frame. (E) For the theta motion target, the Gaussian window and the random dots texture moved at the same speed but in the opposite direction.

The participants were seated 70 cm in front of a CRT monitor (22-inch, Diamond Pro 2070SB, Mitsubishi, refresh rate of 100 Hz, background mean luminance 60 cd/m^2^) with head stabilized by a chin rest and a forehead restraint. Eye position signals from the right eye were calibrated by requiring the participants to fixate a target spot (diameter of 0.3 deg) at known horizontal and vertical eccentricities in binocular viewing condition. The visual stimuli and target motion were generated by Psychophysics Toolbox extensions on MATLAB (Mathworks, MA). Smooth pursuit was produced by a step-ramp paradigm [35] with a constant speed of 18.5 deg/s. The pursuit stimuli (diameter of 2 deg) were random dot fields (each dot, 5 x 5 pixels) whose contrast was modulated by a Gaussian window with a space constant of 20 pixels. Dots had a density of 50% and dot lifetime was equal to presentation duration (1000 ms). We used five types of visual motion target. For the first-order motion target, both the Gaussian window and random dots texture moved in the same direction at the same speed (Fig 1A). For the first-order+ motion target, the Gaussian window and random dots texture moved in the same direction but random dots texture moved at twice the speed of the Gaussian window (Fig 1B). For the second-order static motion target, the Gaussian window moved over a static-background that consists of random dots texture (Fig 1C). For the second-order dynamic motion target, the Gaussian window moved over a dynamic-background where random dots texture was replaced on every frame (Fig 1D). For the theta motion target, the Gaussian window and the random dots texture moved at the same speed but in the opposite direction (Fig 1E) [36]. The participants first fixated on a central stationary target (diameter of 0.3 deg) that appeared on uniform gray background for 3.0 s and a pursuit target appeared 1.4 deg left or right from the center. The target then started to move either leftward of rightward. The participants were instructed to track a moving target with their eyes. The smooth pursuit eye movement tasks consist of 100 trials as 10 trials were conducted for each direction (20 trials x 5 different visual motion). Ten possible combinations (2 directions x 5 motion targets) were presented by random order.

### Visual motion RT task

The participants were seated in front of a CRT monitor as a smooth pursuit eye movement task with holding buttons in each hand. A central fixation target appeared on uniform gray background for 1.0–1.5 sec and then the target started to move leftward or rightward randomly at a constant speed of 18.5 deg/sec. The participants were instructed to fixate on the stationary target and press one of the buttons corresponding to the direction of target motion as soon as possible once the target starts to move. Visual motion RT was determined by the time lapse between the onset of target motion and press a button. Five visual motion targets used in smooth pursuit were also applied to visual motion RT tasks (Fig 1). Visual motion RT tasks were continued until 60 successful trials were obtained. Then, 6 successful trials were conducted for each direction (12 trials x 5 different visual motion). The leftward and rightward directions for smooth pursuit were randomized. Although it was theory possible that one direction was presented six times in a row, such an order bias was not observed. To reduce an influence of directional prediction on visual motion RT, the last trial (6th trial) on each direction was removed for following data analysis. The five blocks were set by random order. Visual motion RT of each visual motion condition was determined by a mean value of all the trials for each condition regardless of the target motion direction. The protocol of visual motion RT task was set with reference to the study by Ono and colleagues [21].

### Light on RT task

To clarify the properties of visual motion RT, a light on RT task without visual motion was conducted as the control task. After a central fixation target was presented on uniform gray background for 1.0–1.5 s, the target appeared 2.8 deg left or right side of central position. The participants pressed one of the buttons corresponding to the target position as soon as possible once the target appears. The presented target consisted of a Gaussian window and a stationary random dots texture as well as the visual motion RT task. The sides the target appeared were randomized. Twelve successful trials were conducted and then the mean value was calculated from all the successful ten trials except for the last trial on each side.

### Data collection and analysis

Eye movements during smooth pursuit were detected using a video-based eye tracking system [37]. Eye position signals were digitized at 1 kHz with 16-bit precision using CED-Micro 1401 hardware (Cambridge Electronic Designs, Cambridge, England). Eye velocity and acceleration were generated by digital differentiation of the position arrays using a central difference algorithm in MATLAB (Mathworks, MA, US). Velocity and acceleration data were filtered using an 80-point finite impulse response (FIR) digital filter with a passband of 80 Hz. Saccades were identified by velocity of 30 deg/s or acceleration of 1000 deg/s^2^, and were replaced with NaN before averaging data. The remained eye velocity traces were aligned on the onset of target motion and averaged from 20 trials (summation of both direction) in each condition. Pursuit initiation during step-ramp tracking was taken as the time that average eye velocity reached > 3 times standard deviation (SD) above the pretrial values during fixation. Initial acceleration on smooth pursuit was determined as a mean value in the first 100 ms period of smooth pursuit [38, 39]. Latency on pursuit initiation was defined by the time lapse between the onset of target motion and onset of smooth pursuit.

### Statistical analysis

To examine the influences of visual motion targets (the first-order motion, first-order+ motion, second-order static motion, second-order dynamic motion and theta motion) on initial eye acceleration, pursuit latency and visual motion RT, a one-way analysis of variance (ANOVA) with repeated measures was performed. Significant results on the ANOVA were followed up with post-hoc multiple comparison with Bonferroni correction. Effect size of ANOVA were reported as partial *η*^2^. Partial *η*^2^ were defined as for small when partial *η*^2^ < 0.1, moderate when 0.1 ≤ partial *η*^2^ < 0.08, large when 0.08 ≤ partial *η*^2^ < 0.2, and very large when 0.5 ≤ partial *η*^2^, respectively.

To evaluate the relationship between visual motion RT and pursuit initiation (i.e., initial eye acceleration and pursuit latency), we used within-subjects correlation coefficient which is a method to focus on the changes of variable within each participant, described by Bland and Altman [40]. This method treats individual participant as a categorical factor and applies it to multiple regression. A significant within-subjects correlation coefficient indicates that both pursuit initiation and visual motion RT are altered conjunctively regardless of potential difference in the individuals regarding smooth pursuit and motion perception. In addition, we calculated the mean values of visual motion RT, pursuit latency and initial eye acceleration across all the motion types and then tested the relationship between visual motion RT and pursuit initiation by Pearson’s correlation coefficient. Moreover, Pearson’s correlation coefficient between the light on RT and averaged pursuit latency/acceleration across all the visual motion was calculated. All statistical tests were executed with a significance level of 0.05 and conducted by IBM SPSS software version 26 (SPSS Inc., IL, US).

## Results

Unless noted otherwise, data are presented as mean ± SD.

### Smooth pursuit eye movement

**Fig 2:**
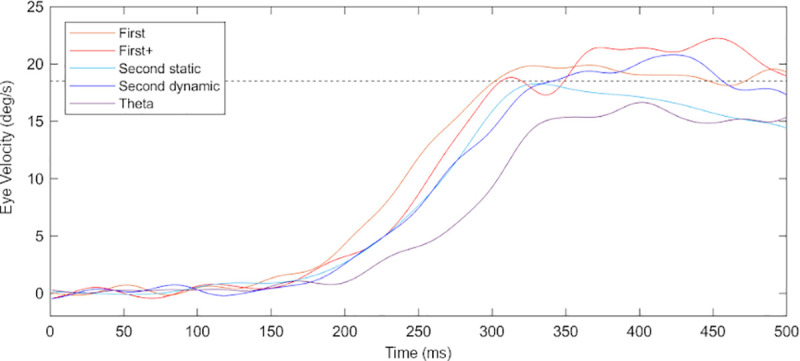
Typical traces of pursuit eye velocity for each target motion from a representative participant. Each trace indicates the mean value of de-saccadic eye velocity of 20 trials for each motion stimulus. Horizontal dashed line indicates the target velocity (18.5 deg/s). Pursuit latency of this participant for five target motion were 142 ms for first-order motion, 162 ms for first-order+ motion, 170 ms for second-order static motion, 179 ms for second-order dynamic motion, and 215 ms for theta motion. Initial eye acceleration of this participant for five target motion were 110.8 deg/s^2^ for first-order motion, 107.5 deg/s^2^ for first-order+ motion, 85.6 deg/s^2^ for second-order static motion, 81.5 deg/s^2^ for second-order dynamic motion, and 58.4 deg/s^2^ for theta motion. Upward deflections show rightward eye motion.

**Fig 3:**
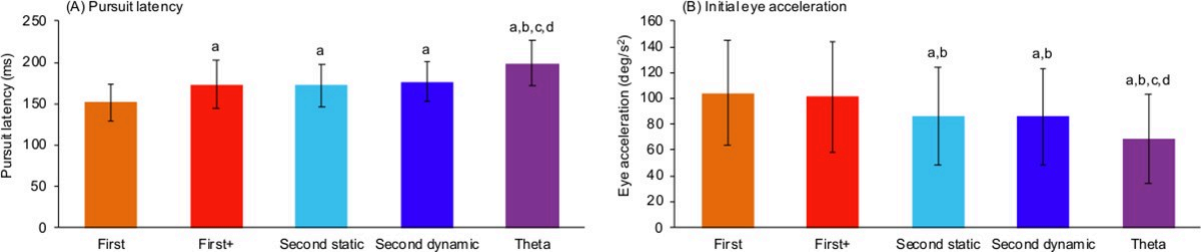
(A) Pursuit latency and (B) initial eye acceleration in different smooth pursuit tasks. The lowercase letters above each bar indicate significant differences (a: vs. first-order; b: vs. first-order+; c: vs. second-order static; d: vs. second-order dynamic). All the significant levels were set at *p* < 0.05.

Fig 2 shows typical traces of eye velocity for each visual motion target from a representative participant. Significant main effects of visual motion targets were observed in both pursuit latency (*F*_4,44_ = 51.39, *p* < 0.01, partial *η*^2^ = 0.82, power > 0.99) and initial eye acceleration (*F*_4,44_ = 13.25, *p* < 0.01, partial *η*^2^ = 0.55, power > 0.99). Post-hoc test showed that pursuit latency for theta motion (199.3 ± 27.1 ms) was significantly longer than the other visual motion targets. Although no significant difference was observed between first-order+ motion, second-order static and dynamic motions, these visual motion tasks yielded longer pursuit latency compared to first-order motion (Fig 3A). Similarly, initial eye acceleration for theta motion (68.6 ± 34.3 deg/s^2^) was smaller than that for the other target stimuli. Furthermore, initial eye acceleration for second-order static and dynamic motions were smaller than first-order and first-order+ motions (Fig 3B).

### Visual motion RT

**Fig 4:**
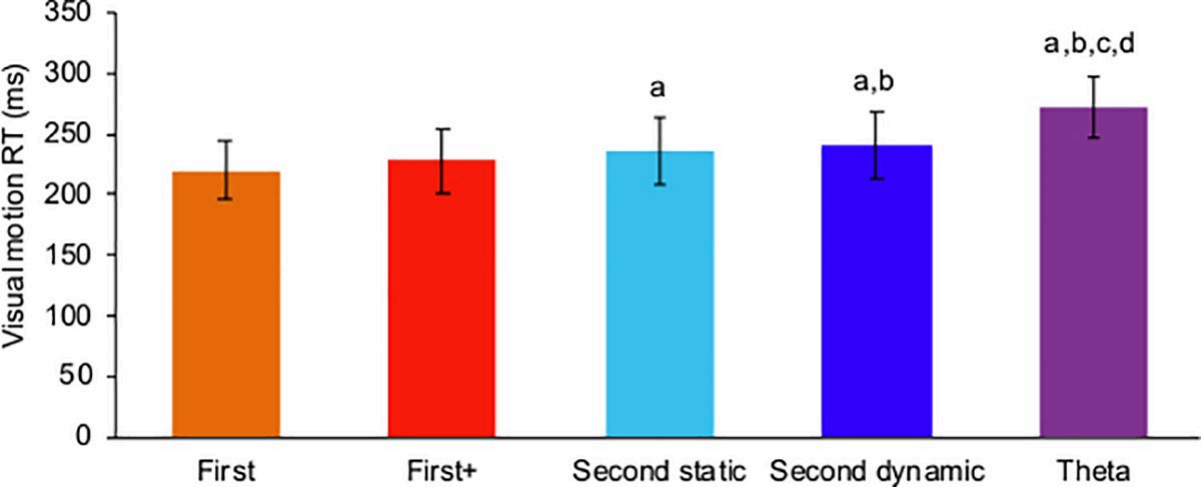
Visual motion reaction time (RT). The lowercase letters above each bar indicate significant differences (a: vs. first-order; b: vs. first-order+; c: vs. second-order static; d: vs. second-order dynamic). All the significant levels were set at *p* < 0.05.

A significant main effect of visual motion target was observed in visual motion RT (*F*_4,44_ = 69.32, *p* < 0.01, partial *η*^2^ = 0.86, power > 0.99) as well as smooth pursuit. A remarkable delay in visual motion RT was yielded by theta motion (272.8 ± 26.4 ms) compared to the other visual motions. The fastest visual motion RT was obtained for first-order motion (220.1 ± 23.9), although statistical difference was not observed between first-order motion and first-order+ motion (Fig 4).

### Relationship between visual motion RT and pursuit initiation

**Fig 5:**
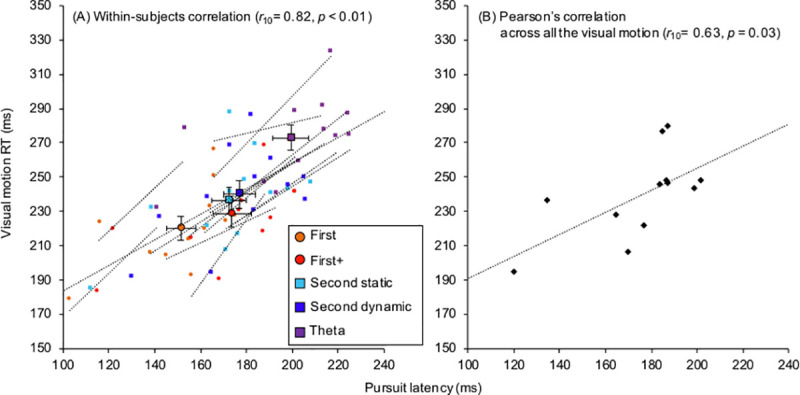
Relationship between visual motion reaction time (RT) and pursuit latency. (A) shows the within-subjects correlation between visual motion RT and pursuit latency. Error bars indicate standard error of the mean (SEM) for each motion stimulus. Open circles and connected dotted lines indicate the data and liner regression of each participant. (B) shows the relationship between these variables by the mean values across all the visual motion.

**Fig 6:**
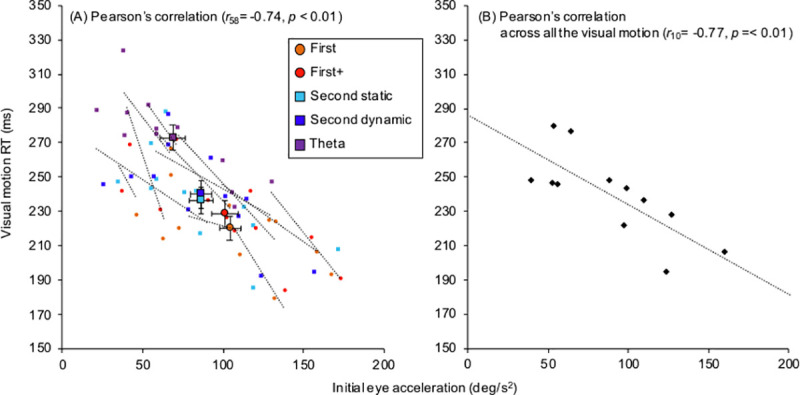
Relationship between visual motion reaction time (RT) and initial eye acceleration. (A) shows the within-subjects correlation between visual motion RT and initial eye acceleration. Error bars indicate standard error of the mean (SEM) for each motion stimulus. Open circles and connected dotted lines indicate the data and liner regression of each participant. (B) shows the relationship between these variables by the mean values across all the visual motion.

Significant within-subjects correlation coefficients were found between visual motion RT and pursuit latency (*r*_10_ = 0.82, *p* < 0.01, Fig 5A) and between visual motion RT and initial eye acceleration (*r*_10_ = -0.72, *p* < 0.01, Fig 6A). Furthermore, Pearson’s correlation coefficients were also observed between visual motion RT and pursuit latency (*r*_10_ = 0.63, *p* = 0.03, Fig 5B) and between visual motion RT and initial eye acceleration (*r*_10_ = -0.76, *p* < 0.01, Fig 6B). Contrast to visual motion RT, the light on RT (214.5 ± 27.2 ms) showed no relationship to pursuit latency (*r*_10_ = 0.33, *p* = 0.29) and initial eye acceleration (*r*_10_ = -0.37, *p* = 0.23).

## Discussion

In this study, we attempted to apply five visual motion stimuli to visual motion RT task to clarify the relevance between visual motion RT and pursuit initiation. Pursuit latency and initial eye acceleration were altered by different visual motion stimuli including first- and second-order motion, which is consistent with previous studies [30–33]. Out of five visual motion targets, theta motion induced a remarkable reduction in pursuit initiation even though pursuit initiation to opposite direction of target movements was not observed as a previous study has reported [30]. Intriguingly, similar changes dependent on motion stimuli were observed in visual motion RT. We then used two types of correlation coefficient (Pearson’s correlation and within-subjects correlation coefficients) to determine the relationship between visual motion RT and pursuit initiation among our participants. The results of the significant within-subjects correlation coefficients between visual motion RT and pursuit initiation indicate that the extent of delay in visual motion RT for second-order motion stimuli is associated with reduction in pursuit initiation dependent on different motion stimuli. Furthermore, the Pearson’s correlation coefficients by the mean values across all the visual stimuli showed that visual motion RT was significantly correlated with both pursuit latency and initial eye acceleration, indicating that individuals who have shorter pursuit latency have faster visual motion RT. Here we discuss underlying mechanisms related to the relationship between visual motion RT and pursuit initiation.

### Neuronal mechanisms of visual motion RT and pursuit

Two types of significant correlation coefficients between visual motion RT and pursuit initiation indicate that both variables are altered in similar manners by different motion stimuli. This was the case even though the velocity of the Gaussian window of visual motion stimuli was constant. It has demonstrated that second-order motion elicits an weaker response in MT and MST to the direction of target motion compared to first-order motion [34, 41] and the target motion defined by theta motion is not explicitly encoded in the neuronal activity in areas MT and MST [31, 33]. The weaker response in these cortical areas is due to low retinal image motion induced by second-order motion. For example, the direction of retinal image motion evoked by random dot motion is not consistent with the direction of window during second-order motion stimuli, which does not evoke explicitly optokinetic nystagmus (OKN) [42, 43]. It is well known that the MT and MST play pivotal roles in pursuit initiation and motion perception [5, 19, 44, 45]. In fact, both pursuit initiation and motion perception become conjunctively changed dependent on targets’ physical feature [16]. Moreover, these parietal cortical areas have reciprocal connection with the frontal eye field (FEF) and frontal pursuit regions, which also play important role in visual motion perception [46–49]. Our results imply that changes in neuronal activity of visual motion sensitive areas by different visual motion stimuli alter visual motion RT as well as pursuit initiation.

Several studies focusing on relationship between steady-state smooth pursuit and direction-discrimination or perceived target velocity have proposed a model where there is an initial common stage of motion processing before two independent processes for smooth pursuit and motion perception [11, 50]. In addition, Mukherjee and colleagues has demonstrated that pursuit initiation and short-timescale motion perception share the dominant source of variation, indicating the existence of a common neuronal pathway for both visual motion processing [51]. The results of this study using visual motion RT support the suggestion that there is a common neuronal pathway involved in both pursuit initiation and motion perception, especially for the speed of visual signal perception and sensory motor processing. Considering that neither pursuit latency nor initial eye acceleration were correlated with the light on RT, it is most likely that the light on RT and visual motion RT are supported by different visual information processing rather than global visual processing.

### Relationship between visual motion RT and pursuit

Our findings showing second-order motion leads to a delay in visual motion RT suggest that the speed of judgement of target direction is a different ability from the direction-discrimination and perceived target velocity tasks. Previous studies have reported that the performances of the direction-discrimination and perceived target velocity tasks are not affected by different visual motion stimuli. For instance, primates correctly perceive the direction of a moving target even though pursuit initiation is inhibited by second-order motion [33]. In human subjects, it has been demonstrated that perceived target velocity is consistent for target motion at or above 4 deg/s regardless of motion stimuli [52], which is termed as form-cue invariance by Albright [41]. Although second-order motion inhibits pursuit initiation due to low retinal image motion [42, 43], it is well known that smooth pursuit can be maintained by not only the retinal image motion signal but also the extraretinal oculomotor feedback where the efference copy and the retinal image motion signal are compared [53–55]. In the light of the close relationship between the steady-state smooth pursuit gain and perceived target velocity [13, 14, 50, 56], reduction in pursuit initiation may not influence deficits in direction-discrimination and perceived target velocity. In contrast, quick visual motion RT needs information regarding the initial phase of target motion rather than steady-state phase, visual motion RT could be tightly related to pursuit initiation.

Previous studies using sinusoidal pattern grating stimulus have reported that the direction of first-order motion can be discriminated at very brief stimulus attendant time, whereas that of second-order motion needs more exposure duration of up to 200 ms [57, 58]. Similarly, visual-motor RT using grating stimuli is shorter when the stimulus is defined by first-order motion than second-order motion [59, 60]. Note that the grating stimulus used in these studies induces OKN but not smooth pursuit since it does not have an explicit target. Although both behaviors are generated by cortical activity involved in retinal image motion, smooth pursuit is driven by the signals on or near the fovea. Single-unit recording studies have identified that MST neurons with the foveal/parafoveal visual receptive fields show no response to visual motion in peripheral visual fields [3, 61]. Therefore, the visual motion RT task used in this study reflects the speed of visual motion perception and sensory motor processing on or near the fovea, whereas the traditional visual-motor RT task using grating stimulus evaluates RT for a large field of vision.

Visual motion RT task has some methodological advantages. The significant relationship between visual motion RT and pursuit initiation indicates that visual motion RT would be an alternative methodology for degerming initial pursuit responses to target motion as more simple methods. A previous study using visual-motor RT by grating stimulus for a large-field of vision defined by first- and second-order motion has reported that this evaluation is highly sensitive to visuomotor anomalies induced by mild traumatic brain injuries [62]. Therefore, visual motion RT task could detect a neuronal anomaly related to the foveal/parafoveal visual receptive fields more specifically. Further study will be needed to clarify the practical utility of visual motion RT using target motion stimuli.

## Conclusion

Our findings expand previous studies regarding the relationship between smooth pursuit and the speed of visual motion perception using a small target. We attempted to determine the relevance between visual motion RT and pursuit initiation using five different visual motion stimuli including first- and second-order motion. Both pursuit initiation and visual motion RT were altered in similar manners dependent on the visual motion stimuli. Furthermore, both Pearson’s correlation and within-subjects correlation coefficients were significant between visual motion RT and pursuit initiation, indicating an ability of visual motion RT is tightly associated with pursuit initiation. This is the first study to clarify that pursuit initiation is associated with the speed of visual motion perception and sensory motor processing. These results support the suggestion regarding the existence of a common neural pathway involved in both pursuit initiation and motion perception.

## References

[1] Krauzlis R. J. (2004). Recasting the Smooth Pursuit Eye Movement System. *J*. *Neurophysiol*. 91, 591–603. 10.1152/jn.00801.2003 14762145

[2] Lisberger S. G. (2010). Visual guidance of smooth-pursuit eye movements: Sensation, action, and what happens in between. *Neuron* 66, 477–491. 10.1016/j.neuron.2010.03.027 20510853 PMC2887486

[3] Ono S. (2015). The neuronal basis of on-line visual control in smooth pursuit eye movements. *Vision Res*. 110, 257–264. 10.1016/j.visres.2014.06.008 24995378 PMC4779051

[4] Newsome W. T., Wurtz R. H., Dursteler M. R., and Mikami A. (1985). Deficits in visual motion processing following ibotenic acid lesions of the middle temporal visual area of the macaque monkey. *J*. *Neurosci*. 5, 825–840. 10.1523/JNEUROSCI.05-03-00825.1985 3973698 PMC6565029

[5] Dursteler M. R., and Wurtz R. H. (1988). Pursuit and optokinetic deficits following chemical lesions of cortical areas MT and MST. *J*. *Neurophysiol*. 60, 941–965. 10.1152/jn.1988.60.3.940 3171667

[6] Osborne L. C., Lisberger S. G., and Bialek W. (2005). A sensory source for motor variation. *Nature* 437, 412–416. 10.1038/nature03961 16163357 PMC2551316

[7] Osborne L. C., Hohl S. S., Bialek W., and Lisberger S. G. (2007). Time course of precision in smooth-pursuit eye movements of monkeys. *J*. *Neurosci*. 27, 2987–2998. 10.1523/JNEUROSCI.5072-06.2007 17360922 PMC2567916

[8] Osborne L. C., Bialek W., and Lisberger S. G. (2004). Time Course of Information about Motion Direction in Visual Area MT of Macaque Monkeys. *J*. *Neurosci*. 24, 3210–3222. 10.1523/JNEUROSCI.5305-03.2004 15056700 PMC2553809

[9] Watamaniuk S. N. J., and Heinen S. J. (1999). Human smooth pursuit direction discrimination. *Vision Res*. 39, 59–70. 10.1016/s0042-6989(98)00128-x 10211396

[10] Beutter B. R., and Stone L. S. (2000). Motion coherence affects human perception and pursuit similarly. *Vis*. *Neurosci*. 17, 139–153. 10.1017/s0952523800171147 10750835

[11] Stone L. S., and Krauzlis R. J. (2003). Shared motion signals for human perceptual decisions and oculomotor actions. *J*. *Vis*. 3, 725–736. 10.1167/3.11.7 14765956

[12] Krukowski A. E., and Stone L. S. (2005). Expansion of direction space around the cardinal axes revealed by smooth pursuit eye movements. *Neuron* 45, 315–323. 10.1016/j.neuron.2005.01.005 15664182

[13] Spering M., Kerzel D., Braun D. I., Hawken M. J., and Gegenfurtner K. R. (2005). Effects of contrast on smooth pursuit eye movements. *J*. *Vis*. 5, 455–465. 10.1167/5.5.6 16097876

[14] Schütz A. C., Braun D. I., Movshon J. A., and Gegenfurtner K. R. (2010). Does the noise matter? Effects of different kinematogram types on smooth pursuit eye movements and perception. *J*. *Vis*. 10, 1–22. 10.1167/10.13.26 21149307 PMC3039843

[15] Van Donkelaar P., Miall R. C., and Stein J. F. (2000). Changes in motion perception following oculomotor smooth pursuit adaptation. *Percept*. *Psychophys*. 62, 378–385. 10.3758/bf03205557 10723216

[16] Braun D. I., Mennie N., Rasche C., Schütz A. C., Hawken M. J., and Gegenfurtner K. R. (2008). Smooth pursuit eye movements to isoluminant targets. *J*. *Neurophysiol*. 100, 1287–1300. 10.1152/jn.00747.2007 18614758

[17] Newsome W. T., Britten K. H., and Movshon J. A. (1989). Neuronal correlates of a perceptual decision. *Nature* 341, 52–54. 10.1038/341052a0 2770878

[18] Salzman C. D., Britten K. H., and Newsome W. T. (1990). Cortical microstimulation influences perceptual judgements of motion direction. *Nature* 346, 174–177. 10.1038/346174a0 2366872

[19] Newsome W. T., and Pare E. B. (1988). A selective impairment of motion perception following lesions of the middle temporal visual area (MT). *J*. *Neurosci*. 8, 2201–2211. 10.1523/JNEUROSCI.08-06-02201.1988 3385495 PMC6569328

[20] Cooper S. A., Joshi A. C., Seenan P. J., Hadley D. M., Muir K. W., Leigh R. J., et al (2012). Akinetopsia: Acute presentation and evidence for persisting defects in motion vision. *J*. *Neurol*. *Neurosurg*. *Psychiatry* 83, 229–230. 10.1136/jnnp.2010.223727 21217160

[21] Ono S., Miura K., Kawamura T., and Kizuka T. (2019). Asymmetric smooth pursuit eye movements and visual motion reaction time. *Physiol*. *Rep*. 7, 1–8. 10.14814/phy2.14187 31353820 PMC6661271

[22] Thorpe S., Fize D., and Marlot C. (1996). Speed of processing in the human visual system. *Nature* 381, 520–522. 10.1038/381520a0 8632824

[23] Hülsdünker T., Strü Der H. K., and Mierau A. (2017). Visual motion processing subserves faster visuomotor reaction in badminton players. *Med*. *Sci*. *Sports Exerc*. 49, 1097–1110. 10.1249/MSS.0000000000001198 28072633

[24] Hülsdünker T., Ostermann M., and Mierau A. (2019). The Speed of Neural Visual Motion Perception and Processing Determines the Visuomotor Reaction Time of Young Elite Table Tennis Athletes. *Front*. *Behav*. *Neurosci*. 13, 165 10.3389/fnbeh.2019.00165 31379535 PMC6659573

[25] Zwierko T., Osiński W., Lubiński W., Czepita D., and Florkiewicz B. (2010). Speed of visual sensorimotor processes and conductivity of visual pathway in volleyball players. *J*. *Hum*. *Kinet*. 23, 21–27. 10.2478/v10078-010-0003-8

[26] Woods D. L., Wyma J. M., Yund E. W., Herron T. J., and Reed B. (2015). Factors influencing the latency of simple reaction time. *Front*. *Hum*. *Neurosci*. 9 10.3389/fnhum.2015.00131 25859198 PMC4374455

[27] Chubb C., and Sperling G. (1988). Drift-balanced random stimuli: a general basis for studying non-Fourier motion perception. *J*. *Opt*. *Soc*. *Am*. *A* 5, 1986–2007. 10.1364/josaa.5.001986 3210090

[28] Cavanagh P., and Mather G. (1989). Motion: the long and short of it. *Spat*. *Vis*. 4, 103–129. 10.1163/156856889x00077 2487159

[29] Ilg U. J., and Churan J. (2010). “Second-order motion stimuli: A new handle to visual motion processing,” in *Dynamics of Visual Motion Processing*: *Neuronal*, *Behavioral*, *and Computational Approaches* (Springer US), 117–138. 10.1007/978-1-4419-0781-3_6

[30] Lindner A., and Ilg U. J. (2000). Initiation of smooth-pursuit eye movements to first-order and second-order motion stimuli. *Exp*. *Brain Res*. 133, 450–456. 10.1007/s002210000459 10985680

[31] Churan J., and Ilg U. J. (2001). Processing of second-order motion stimuli in primate middle temporal area and medial superior temporal area. *J*. *Opt*. *Soc*. *Am*. *A* 18, 2297–2306. 10.1364/josaa.18.002297 11551064

[32] Hawken M. J., and Gegenfurtner K. R. (2001). Pursuit eye movements to second-order motion targets. *J*. *Opt*. *Soc*. *Am*. *A* 18, 2282–2296. 10.1364/josaa.18.002282 11551063

[33] Ilg U. J., and Churan J. (2004). Motion perception without explicit activity in areas MT and MST. *J*. *Neurophysiol*. 92, 1512–1523. 10.1152/jn.01174.2003 15084645

[34] O’Keefe L. P., and Movshon J. A. (1998). Processing of first- and second-order motion signals by neurons in area MT of the macaque monkey. *Vis*. *Neurosci*. 15, 305–317. 10.1017/s0952523898152094 9605531

[35] Rashbass C. (1961). The relationship between saccadic and smooth tracking eye movements. *J*. *Physiol*. 159, 326–338. 10.1113/jphysiol.1961.sp006811 14490422 PMC1359508

[36] Zanker J. M. (1993). Theta motion: a paradoxical stimulus to explore higher order motion extraction. *Vision Res*. 33, 553–69. 10.1016/0042-6989(93)90258-x 8503201

[37] Matsuda K., Nagami T., Sugase Y., Takemura A., and Kawano K. (2017). “A widely applicable real-time mono/binocular eye tracking system using a high frame-rate digital camera,” in *Lecture Notes in Computer Science (including subseries Lecture Notes in Artificial Intelligence and Lecture Notes in Bioinformatics)* (Springer Verlag), 593–608. 10.1007/978-3-319-58071-5_45

[38] Ono S., and Mustari M. J. (2007). Horizontal smooth pursuit adaptation in macaques after muscimol inactivation of the dorsolateral pontine nucleus (DLPN). *J*. *Neurophysiol*. 98, 2918–2932. 10.1152/jn.00115.2007 17804582

[39] Ono S., and Mustari M. J. (2012). Role of MSTd extraretinal signals in smooth pursuit adaptation. *Cereb*. *Cortex* 22, 1139–47. 10.1093/cercor/bhr188 21768225 PMC3328345

[40] Bland J. M., and Altman D. G. (1995). Statistics notes: Calculating Correlation coefficients with repeated observations: Part 1—correlation within subjects. *BMJ* 310, 446 10.1136/bmj.310.6977.446 7873953 PMC2548822

[41] Albright T. D. (1992). Form-cue invariant motion processing in primate visual cortex. *Science (80-*.*)*. 255, 1141–1143. 10.1126/science.1546317 1546317

[42] Lelkens A. M. M., and Koenderink J. J. (1984). Illusory motion in visual displays. *Vision Res*. 24, 1083–1090. 10.1016/0042-6989(84)90086-5 6506473

[43] Harris L. R., and Smith A. T. (1992). Motion defined exclusively by second-order characteristics does not evoke optokinetic nystagmus. *Vis*. *Neurosci*. 9, 565–570. 10.1017/s0952523800001802 1450108

[44] Churchland A. K., Gardner J. L., Chou I. H., Priebe N. J., and Lisberger S. G. (2003). Directional anisotropies reveal a functional segregation of visual motion processing for perception and action. *Neuron* 37, 1001–1011. 10.1016/s0896-6273(03)00145-4 12670428

[45] Hedges J. H., Gartshteyn Y., Kohn A., Rust N. C., Shadlen M. N., Newsome W. T., et al (2011). Dissociation of neuronal and psychophysical responses to local and global motion. *Curr*. *Biol*. 21, 2023–2028. 10.1016/j.cub.2011.10.049 22153156 PMC3241977

[46] Ding L., and Gold J. I. (2012). Neural correlates of perceptual decision making before, during, and after decision commitment in monkey frontal eye field. *Cereb*. *Cortex* 22, 1052–1067. 10.1093/cercor/bhr178 21765183 PMC3328342

[47] Liu L. D., and Pack C. C. (2017). The Contribution of Area MT to Visual Motion Perception Depends on Training. *Neuron* 95, 436–446.e3. 10.1016/j.neuron.2017.06.024 28689980

[48] Raghavan R. T., and Joshua M. (2017). Dissecting patterns of preparatory activity in the frontal eye fields during pursuit target selection. *J*. *Neurophysiol*. 118, 2216–2231. 10.1152/jn.00317.2017 28724782 PMC5626896

[49] Larcombe S. J., Kennard C., and Bridge H. (2018). Increase in MST activity correlates with visual motion learning: A functional MRI study of perceptual learning. *Hum*. *Brain Mapp*. 39, 145–156. 10.1002/hbm.23832 28963815 PMC5725689

[50] Gegenfurtner K. R., Xing D., Scott B. H., and Hawken M. J. (2003). A comparison of pursuit eye movement and perceptual performance in speed discrimination. *J*. *Vis*. 3, 865–876. 10.1167/3.11.19 14765968

[51] Mukherjee T., Battifarano M., Simoncini C., and Osborne L. C. (2015). Shared sensory estimates for human motion perception and pursuit eye movements. *J*. *Neurosci*. 35, 8515–8530. 10.1523/JNEUROSCI.4320-14.2015 26041919 PMC4452555

[52] Gegenfurtner K. R., and Hawken M. J. (1996). Perceived velocity of luminance, chromatic and non-Fourier stimuli: Influence of contrast and temporal frequency. *Vision Res*. 36, 1281–1290. 10.1016/0042-6989(95)00198-0 8711907

[53] Lisberger S. G., Morris E. J., and Tychsen L. (1987). Visual motion processing annd sensory-motor integration for smooth pursuit eye movements. *Annu*. *Rev*. *Neurosci*. Vol. 10, 97–129. 10.1146/annurev.ne.10.030187.000525 3551767

[54] Keller E. L., and Heinen S. J. (1991). Generation of smooth-pursuit eye movements: neuronal mechanisms and pathways. *Neurosci*. *Res*. 11, 79–107. 10.1016/0168-0102(91)90048-4 1656345

[55] Thier P., and Ilg U. J. (2005). The neural basis of smooth-pursuit eye movements. *Curr*. *Opin*. *Neurobiol*. 15, 645–652. 10.1016/j.conb.2005.10.013 16271460

[56] Spering M., and Montagnini A. (2011). Do we track what we see? Common versus independent processing for motion perception and smooth pursuit eye movements: A review. *Vision Res*. 51, 836–852. 10.1016/j.visres.2010.10.017 20965208

[57] Derrington A. M., Badcock D. R., and Henning G. B. (1993). Discriminating the direction of second-order motion at short stimulus durations. *Vision Res*. 33, 1785–1794. 10.1016/0042-6989(93)90169-w 8266634

[58] Ledgeway T., and Hess R. F. (2002). Failure of direction identification for briefly presented second-order motion stimuli: Evidence for weak direction selectivity of the mechanisms encoding motion. *Vision Res*. 42, 1739–1758. 10.1016/s0042-6989(02)00106-2 12127107

[59] Ellemberg D., Lavoie K., Lewis T. L., Maurer D., Lepore F., and Guillemot J. P. (2003). Longer VEP latencies and slower reaction times to the onset of second-order motion than to the onset of first-order motion. *Vision Res*. 43, 651–658. 10.1016/s0042-6989(03)00006-3 12604101

[60] Ledgeway T., and Hutchinson C. V. (2008). Choice reaction times for identifying the direction of first-order motion and different varieties of second-order motion. *Vision Res*. 48, 208–222. 10.1016/j.visres.2007.11.008 18096198

[61] Ono S., and Mustari M. J. (2016). Response properties of MST parafoveal neurons during smooth pursuit adaptation. *J*. *Neurophysiol*. 116, 210–217. 10.1152/jn.00203.2016 27098026 PMC4961761

[62] Piponnier J. C., Forget R., Gagnon I., Mckerral M., Giguère J. F., and Faubert J. (2016). First-and second-order stimuli reaction time measures are highly sensitive to mild traumatic brain injuries. *J*. *Neurotrauma* 33, 242–253. 10.1089/neu.2014.3832 25950948

